# Two-species community design of lactic acid bacteria for optimal production of lactate

**DOI:** 10.1016/j.csbj.2021.11.009

**Published:** 2021-11-09

**Authors:** Maziya Ibrahim, Karthik Raman

**Affiliations:** a Department of Biotechnology, Bhupat and Jyoti Mehta School of Biosciences, IIT Madras, India; b Centre for Integrative Biology and Systems mEdicine (IBSE), IIT Madras, India; c Robert Bosch Centre for Data Science and Artificial Intelligence (RBC-DSAI), IIT Madras, India

**Keywords:** Genome-scale metabolic models, Constraint-based modelling, Metabolic engineering, Cross-feeding, Microbial consortia

## Abstract

Microbial communities that metabolise pentose and hexose sugars are useful in producing high-value chemicals, resulting in the effective conversion of raw materials to the product, a reduction in the production cost, and increased yield. Here, we present a computational analysis approach called CAMP (Co-culture/Community Analyses for Metabolite Production) that simulates and identifies appropriate communities to produce a metabolite of interest. To demonstrate this approach, we focus on the optimal production of lactate from various Lactic Acid Bacteria. We used genome-scale metabolic models (GSMMs) belonging to *Lactobacillus*, *Leuconostoc,* and *Pediococcus* species from the Virtual Metabolic Human (VMH; https://vmh.life/) resource and well-curated GSMMs of *L. plantarum* WCSF1 and *L. reuteri* JCM 1112. We analysed 1176 two-species communities using a constraint-based modelling method for steady-state flux-balance analysis of communities. Flux variability analysis was used to detect the maximum lactate flux in the communities. Using glucose or xylose as substrates separately or in combination resulted in either parasitism, amensalism, or mutualism being the dominant interaction behaviour in the communities. Interaction behaviour between members of the community was deduced based on variations in the predicted growth rates of monocultures and co-cultures. Acetaldehyde, ethanol, acetate, among other metabolites, were found to be cross-fed between community members. *L. plantarum* WCSF1 was found to be a member of communities with high lactate yields. *In silico* community optimisation strategies to predict reaction knock-outs for improving lactate flux were implemented. Reaction knock-outs of acetate kinase, phosphate acetyltransferase, and fumarate reductase in the communities were found to enhance lactate production.

## Graphical abstract

**Figure:**
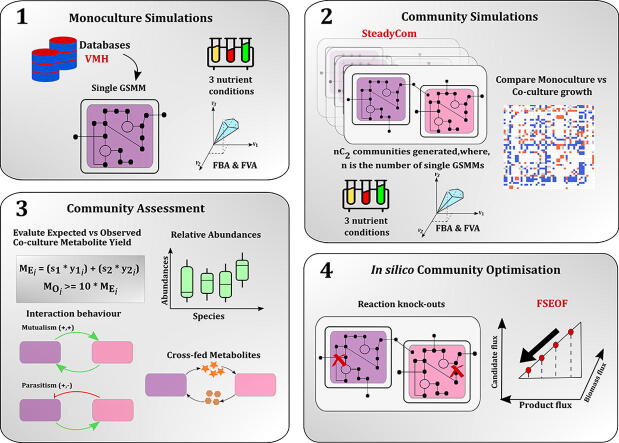

## 1 Introduction

The recent years have witnessed the rising use of co-cultures or microbial communities for the production of various chemicals [1], [2], [3]. In nature, microbes exist in communities, and the use of natural or engineered consortia has many advantages over single strains. One of the critical features of a consortium is the ‘division of labour’ or sharing of metabolic burden between the species. The product of one engineered strain may be transported to another microbe, where it can be further metabolised to the final desired molecule. Co-cultures allow a symbiotic relationship between strains for the utilization of multiple substrates and removal of inhibitory by-products. Some challenges in co-culture studies include compatibility between the strains concerning their growth conditions, such as temperature, pH, and media [4].

Computational modelling of co-cultures is feasible with the use of genome-scale metabolic models (GSMMs). GSMMs of micro-organisms computationally describe the metabolism of an organism through the gene-protein-reaction associations. Progress in the reconstructions of GSMMs has allowed a wide variety of metabolic studies by generating model-driven hypotheses and context-specific simulations by the integration of various omics and kinetic data [5]. GSMMs have been used to predict targets for gene manipulation either through knock-outs or up-and down-regulation, which have resulted in improved production of industrially relevant chemicals from micro-organisms [6], [7]. In an *E. coli* strain (XB201T) producing 0.55 g/L of D-phenyl lactate, knock-outs of *tyrB* and *aspC* genes that were identified as potential knock-out candidates from *in silico* analysis enhanced the production to 1.62 g/L [6].

A number of modelling approaches have been used to study microbial interactions in communities [1], [8], [9], [10]. In particular, constraint-based modelling approaches are very useful to study metabolic interactions between species in a community [54]. Methods to design division of labor in microbial communities have also been developed using GSMMs [11]. In the current study, we present a constraint-based modelling analysis approach called CAMP (Co-culture/Community Analyses for Metabolite Production), which evaluates a set of GSMMs to identify suitable two-species communities that can produce a given metabolite. We demonstrate this approach by analysing GSMMs of selected Lactic Acid Bacteria (LAB) to construct two-species communities and examine their potential for optimal production of lactate.

Lactate is an α-hydroxy carboxylic acid that is chemically reactive and is synthesised to various intermediates such as acrylic acid, 1,2-propanediol, and lactide. Lactide is the building block for producing polylactic acid (PLA) [12]. PLA is a biodegradable biopolymer that finds applications in the biomedical industry to manufacture stents, surgical sutures, soft-tissue implants, etc. [13]. Lactate is also used in the food industry as an acidulant, a preservative, and an emulsifier [12]. The D-isomer is considered harmful to humans in high doses. It can cause acidosis or de-calcification; hence, the L-isomer of lactate is preferred in the food and pharmaceutical industry [14].

Microbial fermentation is an effective route to produce lactate, as optically pure D- or L-lactate can be produced based on the selection of appropriate micro-organisms. LAB can be classified as either homofermentative or heterofermentative, depending on the metabolism of hexoses and pentoses and the production of end products. In homofermentative cases, the sugars are metabolised via the Embden-Meyerhof-Parnas (EMP) pathway, whereas in the heterofermentative case, the phosphoketolase pathway is active [15].

In *Lactobacillus* co-cultures of *L. brevis* and *L. plantarum* with glucose and xylose as substrates and NaOH treated corn stover, high lactate yields of 0.8 g/g was obtained, which is more significant than in monocultures of the same species [16]. *L. rhamnosus* and *L. brevis* were also used in co-culture, and lactate productivity of 0.7 gL^-1^h^−1^ was obtained [17]. Co-culture of *L. pentosus* and genetically engineered *Enterococcus faecalis* produced 3.68 gL^-1^h^−1^ of lactate [18]. A consortium of cellulolytic fungus *Trichoderma reesei* and *L. pentosus* fermented on whole-slurry pre-treated beech wood led to the production of 19.8 g L^-1^ of lactate. *L. pentosus* consumed cellobiose, avoiding inhibition of *T. reesei* cellulase activity, and acetic acid produced from *L. pentosus* was utilised as a carbon source by the fungus [19]. GSMMs of various LAB such as *Lactobacillus reuteri*, *Leuconostoc mesenteroides*, *Lactobacillus plantarum*, *Lactobacillus casei*, *Lactococcus lactis,* and *Streptococcus thermophilus* have been published [20].

We used the CAMP (Co-culture/Community Analyses for Metabolite Production) approach to predict growth rates of LAB species in monoculture and co-culture. We categorised the interactions in LAB communities based on the changes in predicted growth rates, either unidirectional such as commensalism, amensalism, and neutralism, or bi-directional such as mutualism and competition. We analysed the effects of single and multiple nutrient substrates on interaction types between communities. We examined the metabolites that are exchanged between the species of a community. We predicted reaction knock-outs in LAB communities that would improve lactate flux. Overall, our strategy is generic, and it can be applied to identify communities to produce specific metabolites of interest. We postulate that this analysis strategy will benefit metabolic engineering applications that involve microbial communities.

## 2 Results

In this study, we present our workflow for co-culture/community analyses for metabolite production (CAMP). We intend to provide these collective analyses as a clear strategy for the selection of suitable microbial communities for target metabolite production. In this section, we present a brief overview of our approach, followed by its application to identify the most promising co-cultures for producing lactate.

### 2.1 Overview of CAMP (Co-culture/Community Analyses for metabolite Production)

**Fig. 1:**
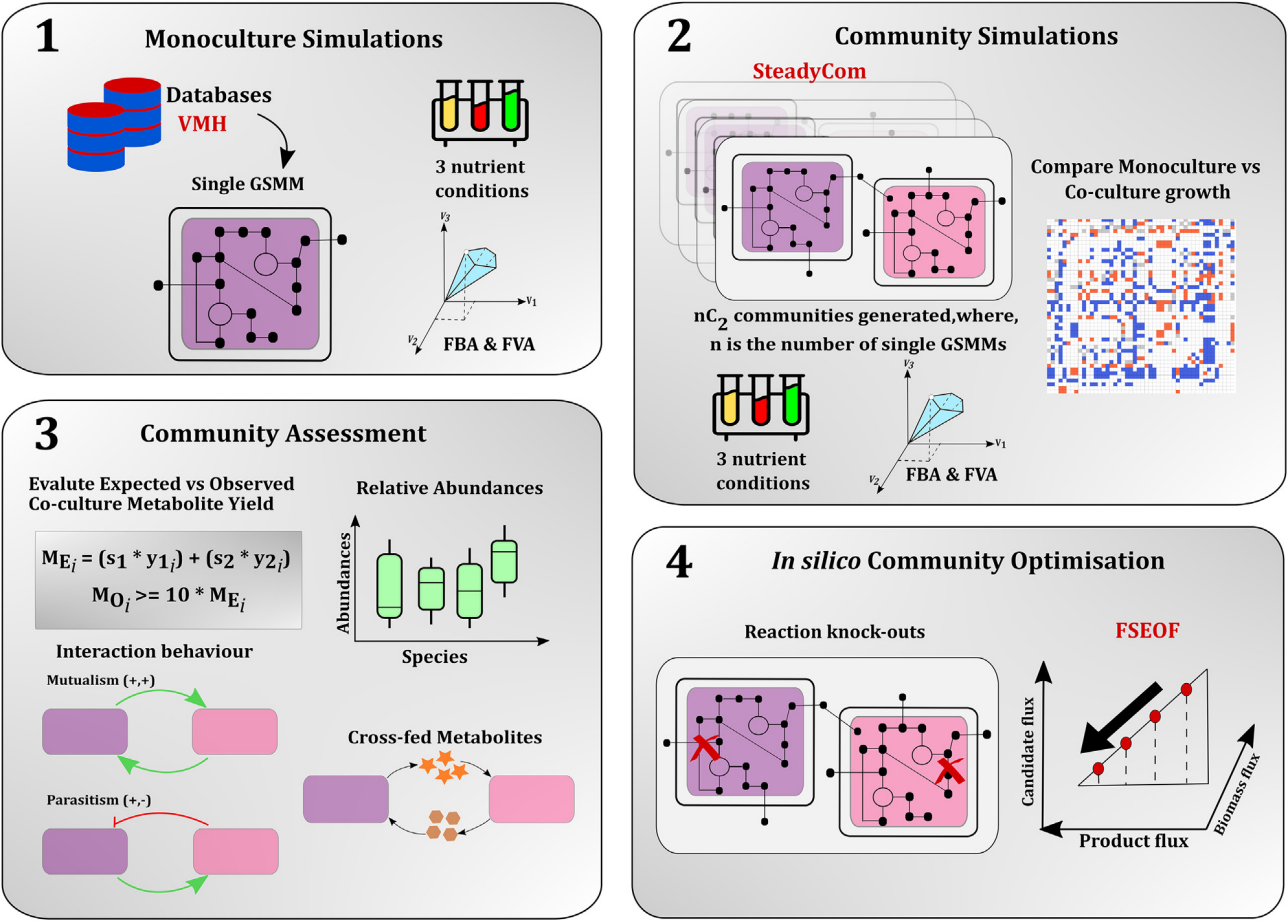
Outline of CAMP (Co-culture/Community Analyses for Metabolite Production) Monoculture and two-species microbial community analyses using FBA and FVA.

Fig. 1 gives an outline of the CAMP workflow. The steps include:

1) Retrieval of microbial GSMMs from databases such as VMH. Each of these GSMMs is simulated in three different nutrient conditions (See Methods). Predicted growth rates and product flux are obtained using flux balance analysis (FBA) and flux variability analysis (FVA). The product yield is computed as the maximum product flux obtained per unit flux of substrate uptake.
2) Two-species communities are created using SteadyCom [10]. Community models are simulated in three nutrient conditions. FBA and FVA are used to predict community growth rates and product yield in the community. Monoculture and co-culture growth rates are compared to identify an increase or decrease in growth when an organism is simulated in the presence of another.
3) Expected product yield in a community is compared to the observed product yield. Details on the calculation of product yield can be found in Methods section. Communities with a 10-fold increase in product yield are regarded as candidate communities for optimal production of the target metabolite. Communities are assessed for their relative abundances, type of interaction behaviour observed, and the cross-fed metabolites.
4) *In silico* community optimisation is performed using FSEOF [21], which enables to shortlist potential reaction knock-outs that will increase product flux in the community. Reaction knock-outs can be from either species in the community.

### 2.2 Growth phenotypes of LAB in monoculture

**Fig. 2:**
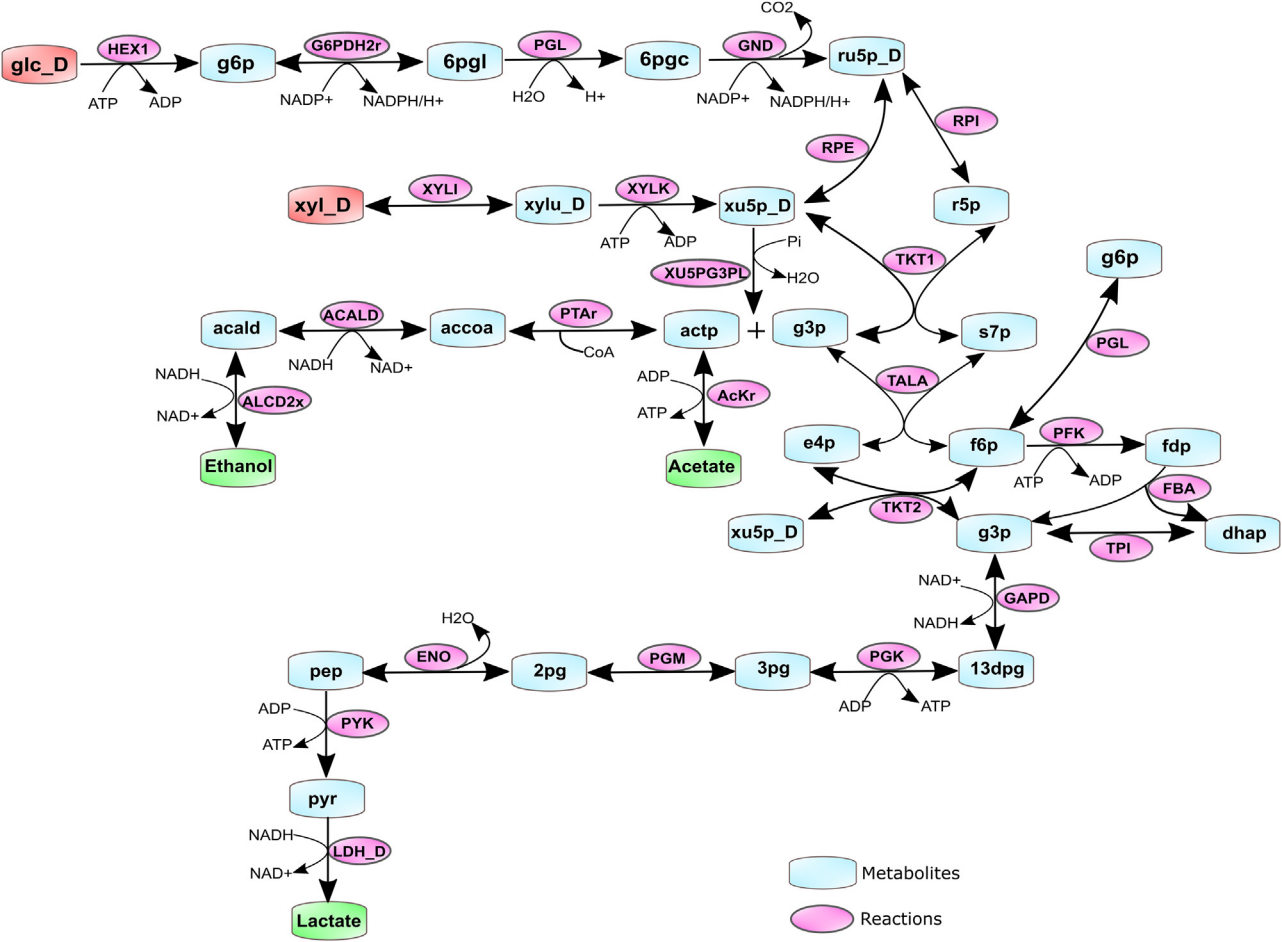
Active pathway reactions with non-zero fluxes in the LAB models when grown in monoculture and co-culture. Glucose and xylose (shaded red) are the primary substrates that are metabolised to the end-products lactate, acetate, and ethanol (shaded green). Metabolite and reaction notations and reaction directionalities are denoted as seen in the LAB GSMMs. (For interpretation of the references to colour in this figure legend, the reader is referred to the web version of this article.)

For all 49 GSMMs, their predicted growth rates in monoculture with glucose and xylose as major carbon sources were computed for the three different nutrient conditions — minimal-nutrient, excess-nutrient, and community-specific nutrient condition (see Methods). The maximal lactate fluxes of each model in all three conditions were also computed. The growth rates of each LAB species in the different nutrient conditions are detailed in S1 Table. It was observed that for all models, the active reactions that had a non-zero flux belonged to the central carbon metabolism, such as Embden-Meyerhof-Parnas (EMP) pathway, pentose phosphate pathway (PPP), and the pentose phosphoketolase (PPK) pathway [22] as seen in Fig. 2. A histogram of predicted monoculture growth rates (S1 Fig) under the three nutrient conditions shows that many species have similar growth rates in all conditions within the range of 0.01 to 0.1 (h^−1^). The highest growth rates (greater than0.3 h^−1^) are observed in the community-specific and excess nutrient conditions.

### 2.3 A significant change in monoculture vs. co-culture growth rates helps segregate communities into six categories

**Fig. 3:**
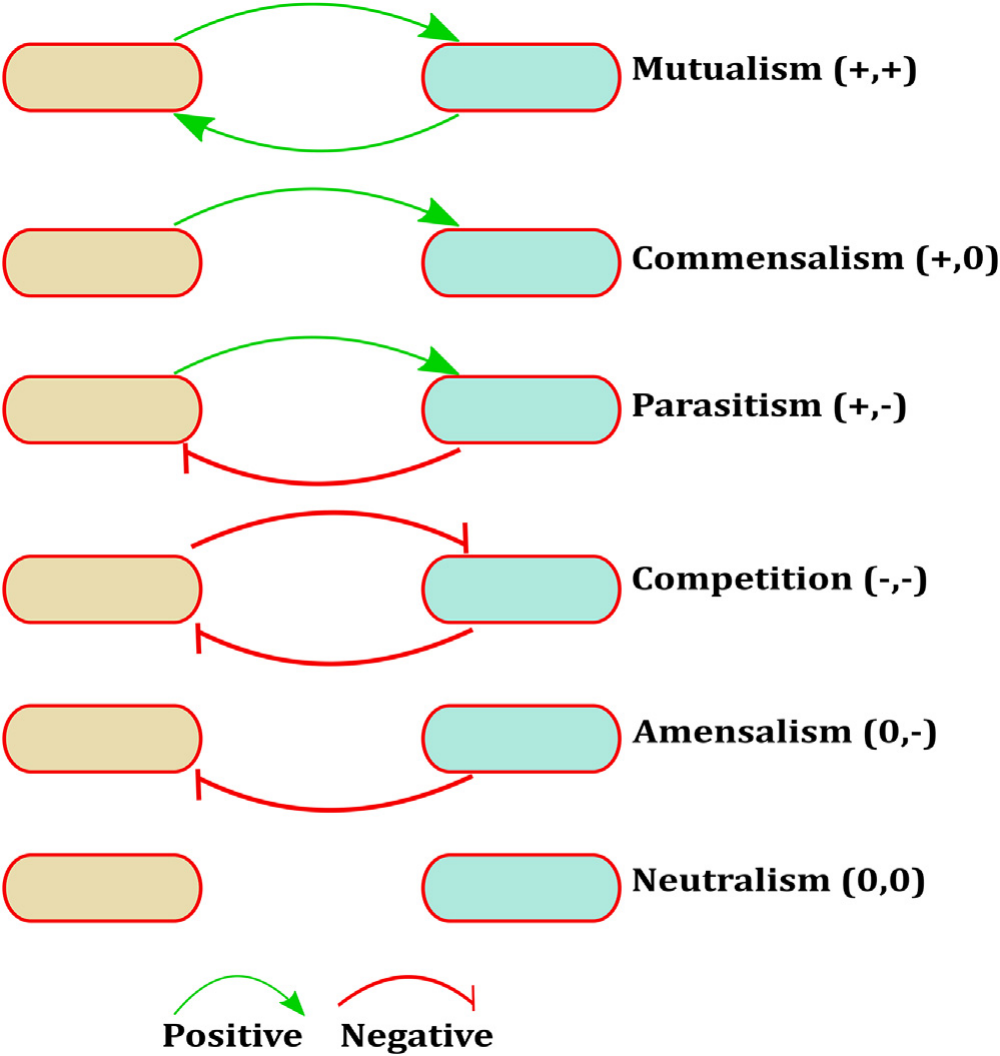
Different interaction types possible between the two-species communities. A positive or negative effect on the growth of the species defines each interaction type.

A difference of 10% in predicted growth rates of the microbes in monoculture versus co-culture has been previously established to be significant [23]. Based on these comparisons, viable LAB communities from each nutrient condition were put into categories as follows: Amensal communities, i.e., one microbe grows slower in the community simulation while the other microbe’s growth rate is unaffected. Competitive communities, i.e., both microbes’ growth, is slower than their monoculture rates. Parasitic communities, i.e., one microbe grows faster in the community simulation while the other microbe grows slower. Neutral communities, i.e., neither microbes’ growth rate was affected upon being paired with the other. Commensal communities, i.e., one microbe, has an increase in growth rate while the other remains unaffected. Lastly, mutualistic communities where both microbes in the pair show an increase in the growth rates compared to their monoculture rates. Fig. 3 depicts the interaction behaviour in communities when each microbe influences the growth of the other, either positively or negatively.

**Fig. 4:**
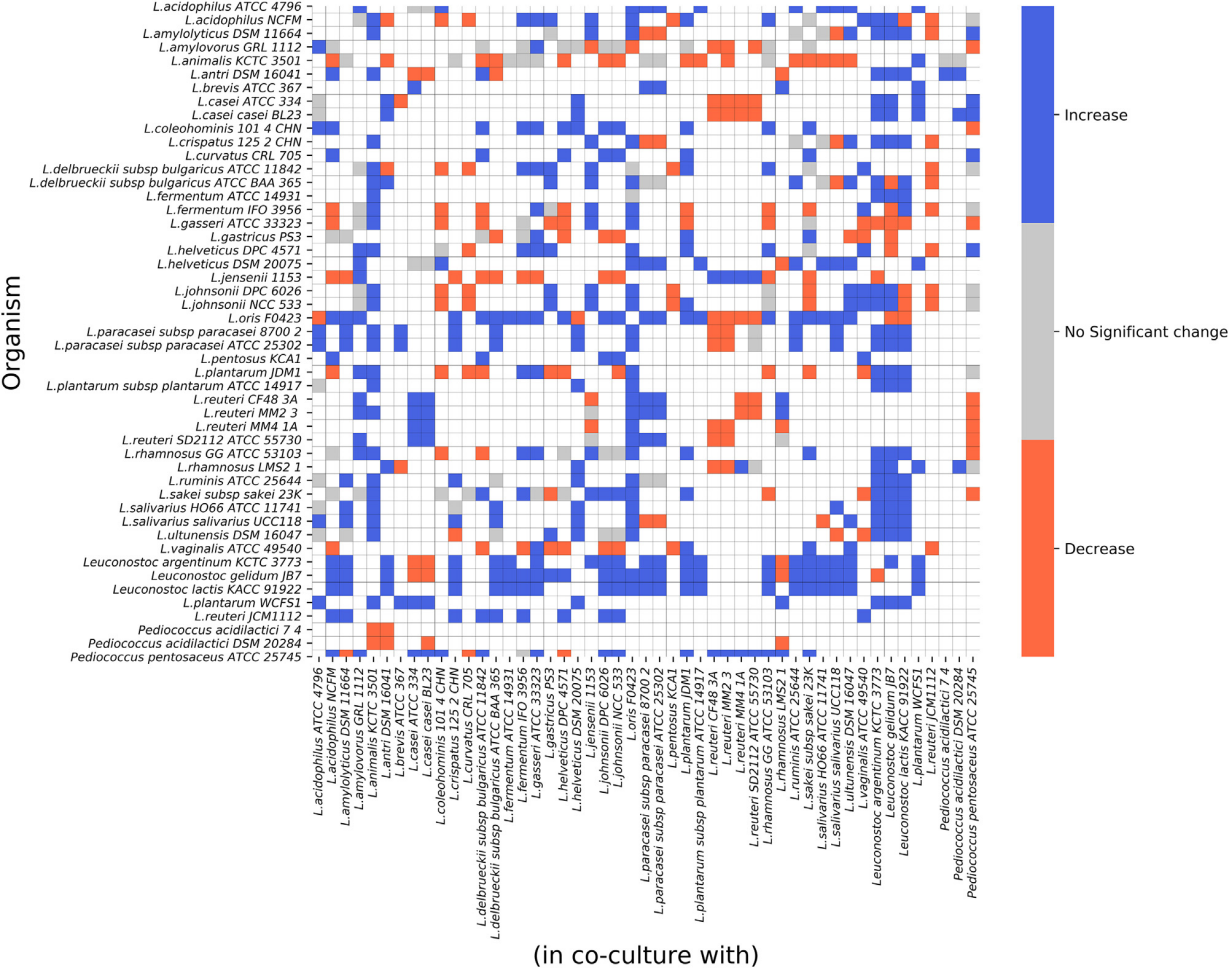
Monoculture vs. co-culture growth rates. The heatmap depicts the change in the growth rate of an organism’s predicted monoculture growth compared to when it is co-cultured with another species under community-specific nutrient conditions. Growth outcomes of 1176 pairwise communities are shown here. A difference greater than 10% of monoculture growth is considered an increase (denoted in blue), whereas lesser than 10% of monoculture growth is regarded as a decrease (denoted in red). 822 non-viable pairs and the diagonal, which represents 49 monocultures, are depicted as white squares. (For interpretation of the references to colour in this figure legend, the reader is referred to the web version of this article.)

In community-specific nutrient conditions, 354 viable pairs out of 1176 were identified, as seen in Fig. 4. Parasitism was the ‘favoured’ interaction type, with 235 pairs out of 354 displaying parasitic behaviour. In minimal nutrient conditions, there were 492 viable pairs. Again, parasitism was dominant in this group, with 224 out of 492 pairs exhibiting parasitism. In contrast, in the excess nutrient condition, from among 338 viable pairs, 215 pairs had amensal behaviour. Parasitism, mutualism, and commensal pairs were not identified in this group. Heatmaps for the minimal and excess nutrient conditions are provided as S2 & S3 Figs. S4-S6 Figs contain heatmaps that depict the absolute values of the predicted growth rates of each species grown in the presence of 48 other species.

### 2.4 Occurrences and relative abundance profiles of the LAB species

The frequency of occurrence of each microbe among the viable communities in each nutrient condition was calculated. *L. oris* and *L. animalis* had the highest occurrences among all *Lactobacillus* species. *Leuconostoc* species were also found to rank higher in the number of occurrences among the viable set, irrespective of the nutrient condition. Each of these microbes was found in at least 20 pairs or more. *Pediococcus* species formed the least number of pairs in the community-specific nutrient condition. *L. pentosus* KCA1 was found to constitute the least number of viable pairs (less than 10) in all nutrient conditions.

**Fig. 5:**
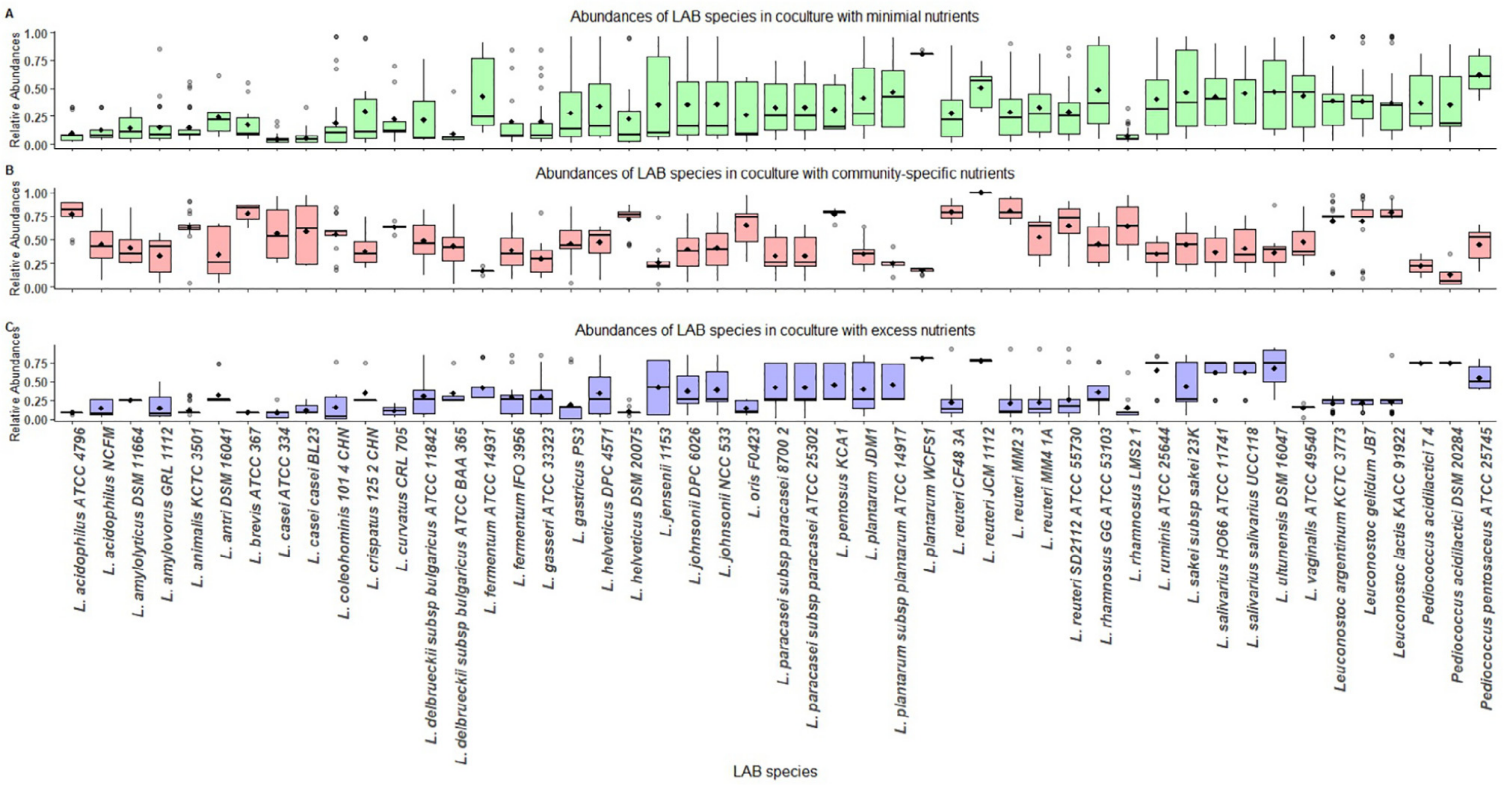
Relative abundance profiles of LAB species in co-culture under different nutrient conditions (A) minimal nutrient condition (B) community-specific condition (C) excess nutrient condition.

The distribution of predicted relative abundances of each microbe when co-cultured under different nutrient conditions are shown in Fig. 5. The abundances were found to vary depending upon the number of viable communities associated with each microbe. Differences were also seen among the nutrient conditions, with most LAB species having a mean abundance of lesser than 0.5 in the excess nutrient condition. *L. oris,* present in many viable communities, had an average abundance of less than 0.25 in the minimal and excess nutrient conditions. In contrast, it had an abundance higher than 0.5 in the community-specific condition. Relative abundances greater than 0.75 were seen among *Leuconostoc* species and some *Lactobacilli* species in the community-specific nutrient condition. This variation in abundance profiles highlights the role of nutrient constraints in driving community behaviour.

### 2.5 Dominant interaction behaviour differs in communities grown with single and multiple substrates

Symbiotic interactions in microbial communities are found to arise more readily though pertubations in the enviroment than genetic alterations, thereby highlighting the role of environments in inducing microbial ecosystems [24].

**Fig. 6:**
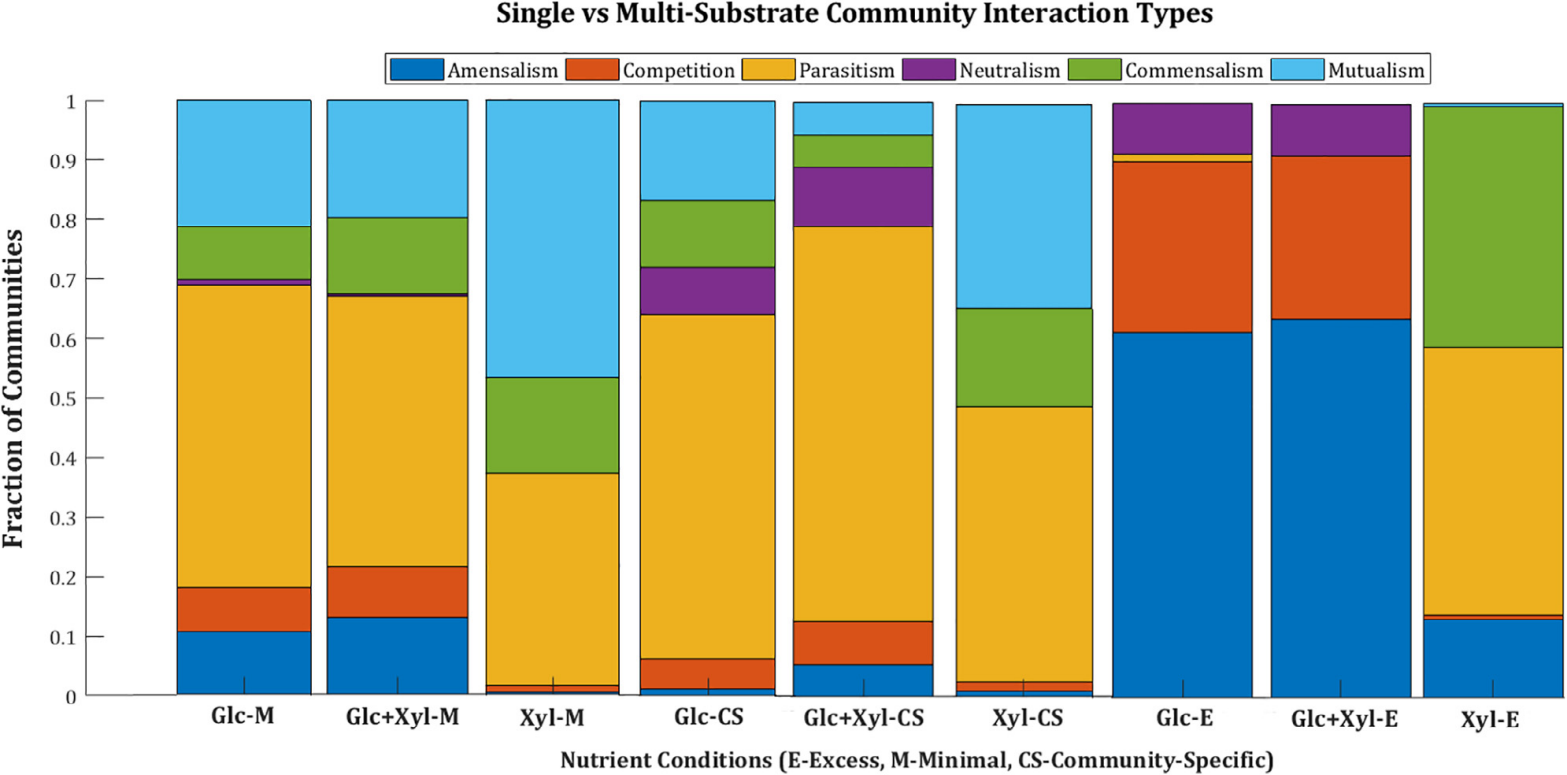
Distribution of the various interaction types between viable pairs in nine different nutrient conditions. The plot shows the fraction of communities with a particular interaction type in each nutrient condition.

To examine if the type of interaction detected in a community is dependent on the number of carbon sources utilised, we simulated the community models for growth on glucose and xylose independently. We compared these findings to when both glucose and xylose are provided as substrates to the communities for growth. Fig. 6 highlights the interaction types observed when glucose or xylose is used as a substrate under different nutrient conditions.

Among the 49 LAB models, only 11 models can metabolise xylose as a sole nutrient source. Mutualistic pairs constituted an average of 40% of viable pairs in the minimal and community-specific conditions with xylose as substrate. The number of mutualistic pairs in xylose-only conditions indicates the rise of an emergent property in the community. Viable pairs with amensalism behaviour are found to be higher in excess nutrient conditions. Parasitism prevailed in both minimal and community-specific nutrient conditions irrespective of the presence of a single or multi-substrate. As all 49 organisms are capable of metabolising glucose, some competitive behaviour is observed primarily in glucose-only excess conditions. Whereas, in xylose-only conditions, competition is almost absent, with only a maximum of three viable pairs exhibiting competition.

### 2.6 Cross-fed metabolites in the LAB communities

Cross-feeding is an emergent property in the assembly of microbial communities, such cross-feeding networks may stabilise competition within related species for the carbon source provided [25]. Metabolic cooperation aided by metabolic exchanges or cross-feeding of amino acids and sugars have been identified as a key driver of co-occurrence in microbial communities of diverse habitats [26]. Cooperative communities which are metabolically dissimilar are found to have higher cross-feeding potential [27]. Metabolic secretions that do not alter the fitness of the species are termed as costless secretions. Such secretions are found to promote interspecies interactions in microbial ecosystems [28].

In CAMP, a metabolite was considered cross-fed if it was secreted (i.e., the flux of the exchange reaction for the particular metabolite was positive) into the community compartment (u) by one organism and taken up (i.e., the flux of the exchange reaction of the metabolite was negative) by the other organism in the community. A threshold of 2 mmol/gDW/h was used to determine all such cross-fed metabolites for the viable communities in each nutrient condition. Fourteen metabolites were cross-fed between the LAB communities. The most widely cross-fed metabolites across all viable communities were acetaldehyde, ethanol, acetate, and formate. Lactate was also found to be cross-fed between 35% of communities across different nutrient conditions. Metabolites such as amino acids and inorganic compounds that were provided as a part of the growth nutrient media were not considered as cross-fed. Each community model exchanged varied sets of metabolites depending on the nutrient condition it was simulated in. We checked whether the cross-fed metabolites are specific to any interaction type and found that the metabolites are common to all interaction types. The fraction of metabolites cross-fed in cooperative communities with mutualistic, commensal, and neutral interactions is higher than in communities that exhibit parasitic and competitive behaviour. S2 Table has the list of cross-fed metabolites in each interaction type.

### 2.7 Evaluating the performance of communities based on growth and lactate yield

We evaluated the performance (see Methods) of the community models in two scenarios. In the first set of simulations, lactate was not allowed to be cross-fed between the community members. In the second case, one organism in the pair is designated as the primary consumer of the substrates glucose and xylose, thereby creating a dependence of the second organism on the first for growth and vice-versa. Community pairs that retained their viability in the two test scenarios were deemed fit for further community strain optimisation strategies. This performance test was carried out in all three nutrient conditions. Forty community pairs were common in two nutrient conditions, community-specific nutrient uptake and minimal nutrient uptake. Seven LAB communities were unique to the excess nutrient condition. Each of these pairs had an observed lactate yield 10-fold higher than the expected lactate yield of the community (S3 Table).

### 2.8 Glucose fermenters have a higher lactate yield than communities where both xylose and glucose is utilised

For grading the community pairs based on both their growth rate and product yield, the biomass, and lactate flux values were normalised (min–max normalization). Upon normalisation, the best pairs were identified. A detailed list of all communities is found in S3 Table. Each of the top six pairs shared an organism, namely, *L. plantarum* WCFS1, which is coupled with two strains of *L. casei*, *L. rhamnosus* LMS2, *L. animalis* KCTC 3501, *Leuconostoc argentinum,* and *Leuconostoc lactis*.

To determine why specific communities fare better in lactate production, we examined the changes in the reaction fluxes of these communities as well as the cross-fed metabolites. We found no significant association between the cross-fed metabolites and lactate yields. However, by comparing reaction fluxes from a random set of four communities between the two groups, i.e., high-lactate producers and low-lactate producers, we find some reactions have a five-fold increase in fluxes in the high-lactate group (S4 Table). These reactions include glyceraldehyde 3-phosphate dehydrogenase, triose-phosphate isomerase, fructose bisphosphate aldolase, fumarate reductase, transaldolase, and phosphoenolpyruvate carboxylase. The reactions belong to pathways associated with carbohydrate metabolism and hence may be instrumental in regulating the lactate flux.

Contrary to expectations, in the best-performing pairs, both the organisms are not capable of utilising glucose and xylose together. Only the *Leuconostoc* species can metabolise both glucose and xylose, while the remaining organisms are glucose fermenters. The metabolic distances (Jaccard distances) between the GSMMs in the best-performing pairs were calculated (see Methods) using reaction lists from each model. The top-ranked pairs had a Jaccard distance greater than 0.7, indicating that they had less than 30% of their reactions in common, and therefore, distinct metabolic capabilities. Besides, all the high-lactate-producing communities displayed either commensal, mutualistic, or neutral interaction behaviours in the three different nutrient conditions. This suggests that metabolic complementarity and compatibility between the organisms are necessary for the stability of a community.

### 2.9 Elimination of reactions from competing pathways provide an enhanced lactate flux in the LAB community

Due to the paucity of methods that are designed to predict gene or reaction knockouts in microbial communities, we adapted existing strategies such as FSEOF (Flux Scanning based on Enforced Objective Flux), which were designed for single-species microbial models, to predict reaction knock-outs in the LAB communities. Based on the FSEOF approach (see Methods), we could predict suitable reaction knock-outs in six LAB community models that improved lactate flux compared to the flux obtained in the wild-type community. These communities each had one organism from the *Leuconostoc* genus, capable of fermenting both glucose and xylose. These community species are heterofermentative, i.e., they are capable of the production of mixed organic acids such as ethanol, formate, and acetate in addition to lactate. Among the predicted knock-out targets, the reactions with a maximum increase of lactate flux are tabulated in Table 1.

**Table 1:** List of reaction knock-outs that lead to an increased lactate flux in different LAB communities.

Reaction ID	Reaction Name	Reaction Formula
ACKr	acetate kinase	acetate + ATP ⇔ acetyl-phosphate + ADP
PTAr	phosphotransacetylase	acetyl-CoA + phosphate ⇔ acetyl-phosphate + CoA
PFL	pyruvate formate lyase	pyruvate + CoA ⇔ acetyl-CoA + formate
FRD	fumarate reductase	fumarate + ubiquinol-8 ⇔ succinate + ubiquinone-8
RPE	ribulose 5-phosphate 3-epimerase	ribulose 5-phosphate ⇔ xylulose 5-phosphate
XU5PG3PL	D-xylulose 5-phosphate D-glyceraldehyde-3-phosphate-lyase	xylulose 5-phosphate + phosphate → acetyl-phosphate + glyceraldehyde 3-phosphate + H_2_O

As evident from these reactions, routes towards the production of other acids, such as acetate, formate, and succinate, are impeded to allow higher flux towards reactions leading to the biosynthesis of lactate. The details of predicted reaction knock-outs in each community model and the equivalent lactate flux observed in that community upon deletion are provided in the S5 Table.

Our findings using this approach for microbial communities concur with experiments observed in literature where deletion of the genes counterpart to these reactions has increased the lactate yield from monocultures of various micro-organisms. An engineered strain of *Enterobacter aerogenes* ATCC 29,007 with the phosphate acetyltransferase (*pta*) gene deletion was found to have a higher L-lactate yield by utilization of mannitol [29]. *Escherichia coli* K12 strain MG1655 has been engineered by the inactivation of the pyruvate-formate lyase (*pflB*) and fumarate reductase (*frdA*) gene to increase the yield of D-lactate from glycerol [30]. A single-gene knock-out of the *pflA* gene in the *E. coli* BW25113 strain has proven to improve D-lactate production from glucose [31]. In *Saccharomyces cerevisiae*, the deletion of D-ribulose-5-phosphate 3-epimerase (RPE1) induces the simultaneous utilization of xylose and glucose [32]. Gene knock-outs are an essential metabolic engineering strategy employed for overcoming barriers of carbon catabolite repression for the co-utilization of carbon sources by microbes [33], [34]. Therefore, we hypothesise that to design efficient microbial communities, appropriate gene knock-outs from either one or both the organisms in a co-culture will enhance the co-utilization of mixed carbon substrates and improve product yield. In this regard, *in silico* approaches as described above will aid in making informed decisions for knock-out experiments.

## 3 Discussion

Lactate synthesis through bacterial fermentation methods is of great importance for improving the compound’s availability and aiding the production of lactate derivatives with high industrial value. While several computational approaches to study microbial communities have emerged in recent years [8], [10], [35], [36], there is still no rigorous methodology to systematically choose a co-culture for optimal production of industrially relevant metabolites, such as the production of lactate. In this study, we report CAMP (Co-culture/Community Analyses for Metabolite Production), an analysis approach to systematically screen multiple candidate communities on multiple substrates under different growth conditions and rank the best-performing communities that will most likely succeed in laboratory experiments. Our approach utilises emerging computational methods with GSMMs in the context of microbial communities of LAB. In pursuit of an ideal two-species community for lactate production, we established a framework where community growth is the objective. The community model is tested for growth on two primary carbon sources, glucose and xylose. Screening of viable communities based on predicted growth and lactate yield further enabled comparison between monoculture and co-culture states. Communities were labelled with specific interaction behaviours because of the changes observed in growth rates. The results obtained elucidated the role of single or multi-substrates for the prevalence of a particular interaction type in the communities. A change in nutrient condition revealed differences in the interaction behaviours of the communities, but this did not influence the results of the top-ranked communities based on lactate yield*.*

In our analysis, the community model of *L. delbrueckii* subsp. *delbrueckii bulgaricus* and *L. paracasei* subsp. *paracasei* was shown to have a 58% higher observed lactate yield than the expected yield. This community was previously observed in experiments to have a lactate yield of 38 g/L from cassava bagasse hydrolysate [37]. Our analysis also showed communities of *L. brevis* ATCC 367 with *L. plantarum* and *L. pentosus* to be viable. In experimental work by Zhang et. al, *L. brevis* ATCC 367 and a related *L. plantarum* ATCC 21,028 species was found to produce greater lactate in coculture than in monoculture, with a yield of 0.8 g/g from poplar hydrolysate [16]. Garde et. al, have co-inoculated *L. brevis* and *L. pentosus* with hemicellulose hydrolysate and have observed a lactate yield of 0.6 g/g sugar and complete substrate utilisation [38]. The community comprising of *L. casei* ATCC 334 and *L. plantarum* WCFS1 was considered as the best-performing pair. These species have been used independently in industrial applications as starter cultures. *L. plantarum* is found in many ecological niches and is one of the model organisms in LAB research [39]. The GSMM of *L. plantarum* was one of the first reported GSMMs from the LAB species [40]. The presence of *L. plantarum* in the top-ranked pairs in our study reiterates the compatibility of this microbe with other LAB species and its utility for lactate production. Other *L. plantarum* and *Leuconostoc* species are used as co-cultures for the fermentation of Chinese sauerkraut [41]. *L. rhamnosus* strains have been co-cultured with *Saccharomyces cerevisiae* for enhanced exopolysaccharide production [42]. *Pediococcus acidilactici* species have been co-cultured with *L. delbrueckii* species for pediocin production in milk [43].

Highly efficient micro-organisms are required to meet the industrial standards for lactate production. This can be achieved through perturbation, i.e., the addition or deletion of genes that enhance the community’s capability to produce lactate. To address this aspect, we undertook an *in silico* strain optimisation approach using FSEOF to predict reactions that can be deleted to improve product flux. The results we observed were encouraging as they were in accordance with previously published experiments where gene deletion was utilised to enhance lactate yield in monocultures of different micro-organisms. These results also allude that gene knock-outs identified in monoculture can be extended to microbial communities as well. The gene knock-outs can be from one or both organisms in a co-culture. Such insights can be used for developing strain optimisation algorithms specifically for microbial communities. Co-cultures and communities of LAB can provide a significant advantage over the engineering of monocultures. With our framework, we have predicted LAB communities, which are useful candidates to produce lactate. These predictions form a ready shortlist for experimental validation. Our workflow can be extended to communities of larger sizes as well, although the increase in combinatorial complexity will also demand an increase in computational cost. The algorithm, SteadyCom has been originally designed to predict species abundance in densely populated microbial communities such as the human gut. In CAMP, SteadyCom has been applied to simulate two-member synthetic communities that are assumed to coexist in an experimental setup for biotechnological uses. Static FBA based algorithms such as SteadyCom have some limitations that may impose ‘forced altruism’ on individual species to produce metabolites for other community members before optimisation of its fitness objective if this can, in turn, maximise the community-level objective function value [44].

Another caveat of this study is the dependence on the quality of the GSMMs used. The biochemical pathways to produce the metabolite of interest should also be well defined in the GSMMs. Nevertheless, as newer, more accurate reconstructions emerge, they can be used in our approach to present more accurate insights into the compatibility and interactions between organisms to choose the best possible community for a given application. Our approach provides a ready framework for integrating additional experimental data arising from transcriptomics studies or ^13^C metabolic flux analyses to constrain the models better and improve the accuracy of the predictions.

In sum, we have presented a systematic workflow for the careful screening and analysis of many microbial co-cultures to produce the desired metabolite. Our method examines these co-cultures across growth conditions and across multiple substrates to identify the most promising candidates for experimental validation. Computational approaches, as presented in this study, can provide additional flexibility and valuable insights towards informing the selection of microbial co-cultures for metabolic engineering.

## 4 Methods

### 4.1 GSMMs

The Virtual Metabolic Human (www.vmh.life) repository was used for retrieving 47 Lactic Acid Bacteria GSMMs. Models (AGORA version 1.03) of *Lactobacillus*, *Leuconostoc,* and *Pediococcus* species were obtained [45]. Previously curated and published GSMMs of *L. plantarum* WCSF1 and *L. reuteri* JCM 1112 were also used to construct the synthetic communities of LAB [20], [40]. A list of all 49 GSMMs used in this study is tabulated in the S1 Table. Three models from VMH, namely, *L. amylolyticus*, *L. crispatus,* and *L. delbrueckii* subsp. *bulgaricus* ATCC BAA 365 did not have the necessary exchange and transport reactions for glucose. We added glucose exchange and transport reactions to these models based on evidence from literature suggesting their capability to metabolise glucose [46].

### 4.2 Creation and growth simulations of two-species communities

We generated all possible pairwise combinations of the 49 species to yield 1176 synthetic LAB communities and simulated them using SteadyCom [10], a constraint-based modelling method for the creation and steady-state flux-balance analysis (FBA) of microbial communities. SteadyCom performs a community FBA by computing the relative abundance of each species with the objective function of maximisation of community growth.

LAB is cultured in laboratories with MRS (deMan, Rogosa, and Sharpe) nutrient media. Analogous growth conditions were simulated *in silico* using nutrient uptake components for LAB models obtained from the KOMODO (Known Media Database) at ModelSEED [47]. All known 20 amino acids were included in this nutrient media. Lignocellulose hydrolysate contains glucose and xylose as significant components. Hence, to mimic this substrate composition, we constrained the lower bounds of glucose and xylose exchange reactions in the community compartment (u) of the models.

Due to a lack of species-specific data for glucose and xylose uptakes, we considered three nutrient conditions: a) a minimal nutrient condition with −1 mmol/gDW/h of glucose and xylose each, b) an excess nutrient condition with constraints of −30 and −10 mmol/gDW/h for glucose and xylose, respectively, and c) finally a community-specific nutrient condition, where we identified the glucose and xylose uptake fluxes at half-maximal growth rates of each model. The lower bounds of the amino acid exchange reactions and other essential components required for model growth were considered as −1 and −1000 mmol/gDW/h, respectively [48]. ATP maintenance constraints for all the LAB models were fixed at 0.36 mmol/gDW/h, as observed in the curated *L. plantarum* WCFS1 and *L. reuteri* JCM 1112 GSMMs. The growth simulations were performed in an anoxic environment, as LAB are anaerobic micro-organisms. Steady-state community growth rates, as well as species abundances, were computed. The goal of this study was to identify LAB communities that are stable and viable; hence the objective function of maximisation of community biomass was deemed suitable to select such communities. All simulations were performed in MATLAB R2018a (MathWorks Inc., USA) using the COBRA Toolbox v3.0 [49] and IBM ILOG CPLEX 12.8 as the linear programming solver.

### 4.3 Categorising communities based on interaction type

Communities were categorised into six interaction types, namely, parasitism, amensalism, commensalism, mutualism, neutralism, and competitive, based on a 10% difference in growth rates of the microbe when grown in co-culture compared to when the bacterium is grown separately [23]. Mutualism and commensalism have a positive effect on community partners, whereas parasitism, competition, and amensalism evoke a negative response on the growth of either partner.

### 4.4 Studying variation in lactate fluxes in a community using FVA

We calculated the maximum lactate produced by a community using FVA on viable communities. FVA computes the flux range of every reaction by minimising and maximising the flux through the reactions [50]. We considered a community viable if each organism in the community had a minimum growth rate of 0.01 h^−1^ or higher [51]. While performing FVA, the biomass reaction in each community was constrained to the maximum community growth rate obtained. SteadyComFVA was used to calculate the maximum flux through the lactate exchange reaction in the community compartment (“EX_lac_D(u)”).

### 4.5 Computing expected vs. Observed lactate yield in each community

The ConYE model proposed by Medlock *et al*. [52] for identifying metabolic mechanisms of interactions within gut microbiota was adapted to our study to calculate and compare the expected and observed lactate yield from each LAB community. The ConYE model identifies metabolites for which the consumption or production behaviour is altered in co-culture. Each strain is assumed to produce or consume a fixed quantity of each metabolite. This assumption is tested by comparing the expected behaviour to the observed co-culture data. The null hypothesis states that the metabolite in co-culture is equal to the predicted amount. Rejecting the null hypothesis implies that the co-culture has caused at least one species to alter the metabolism of the metabolite [46].

With the lactate fluxes identified in monoculture conditions, an estimate of the lactate flux produced in co-culture can be made, considering the substrate utilisation by each species in co-culture. This computed expected lactate yield is compared with the maximum lactate fluxes observed in the community compartment (u) in co-culture.

$$\begin{array}{c} M_{O_{i}}\;\;observed\;metabolite\;yield\\ M_{O_{i}}=\frac{maximum\;metabolite\;flux\;in\;coculture}{\mathrm{Total}\;\mathrm{substrate}\;\mathrm{uptake}}\\ M_{E_{i}}\;=\;(s_{1\;}\times \;y_{1_{i}})\;+\;(s_{2}\;\times \;y_{2_{i}}) \end{array}$$

$M_{E_{i}}$ expected metabolite yield

$s_{1}$ total substrate uptake of species 1 in co-culture

$s_{2}$ total substrate uptake of species 2 in co-culture

$y_{1_{i}}$ the maximum yield of metabolite i in species 1 in monoculture

$$y_{1_{i}}=\frac{maximum\;metabolite\;flux\;of\;species\;1}{substrate\;uptake\;of\;species\;1}$$

$y_{2_{i}}$ the maximum yield of metabolite i in species 2 in monoculture

$$y_{2_{i}}=\frac{maximum\;metabolite\;flux\;of\;species\;2}{substrate\;uptake\;of\;species\;2}$$

If the observed lactate yield of a community is 10-fold higher than the expected yield, i.e. $M_{O_{i}}\geq 10*M_{E_{i}}$ , the community is considered as a candidate pair for lactate production.

### 4.6 Selection of product and growth-efficient communities

Product and growth-efficient communities are defined as communities where a perturbation to the availability of substrates does not affect the viability of the community and the capability to produce lactate. To identify such product and growth-efficient communities, a set of simulations were performed. In the first simulation, the D-Lactate exchange reaction of one organism in the pair was blocked, which prevented cross-feeding of D-Lactate between the community members. Secondly, one organism in the pair was considered as the primary consumer of the substrates, while substrate consumption was blocked in the other organism. Community pairs that retained viability in all simulations were ranked after normalisation (min–max normalisation using the ‘*rescale’* function in MATLAB R2018a) of lactate yields and growth rates.

### 4.7 Metabolic distances of LAB communities

We computed metabolic distances of all LAB models in each community, as described in Magnúsdóttir et al. [53]. The distance is calculated using the Jaccard distance. Metabolic Distance = $\frac{1-\left|R_{i}\cap R_{j}\right|}{\left|R_{i}R_{j}\right|}$ , where *R_i_* is the reaction list from the model *i* and *R_j_* is the reaction list of model *j*. A metabolic distance of 1 indicates that the two models do not share any reactions, whereas a metabolic distance of zero indicates that the models have identical reactions. Among the 1176 LAB communities, 641 had a metabolic distance greater than 0.4 (S6 Table).

### 4.8 Community optimisation and prediction of reaction knock-outs using FSEOF

We performed strain optimisation methods such as the identification of knockout targets in each LAB community that would positively impact lactate production. To this end, we used the FSEOF (Flux Scanning based on Enforced Objective Flux) approach [21]. Using FSEOF, potential reactions to be knocked out were selected based on metabolic flux scanning, which selects fluxes towards product formation. Other constraints used to predict reaction knock-outs included an increase in lactate flux of the mutant community model compared to wild-type and viability (i.e., a growth rate of 0.01 h^−1^ or higher) of both organisms in the community. When the number of reactions obtained from FSEOF was less than or equal to an arbitrary threshold of 30, double deletions were carried out to test all possible knock-out combinations (i.e., a maximum of 435 double deletions) of these reactions. The threshold of 30 reactions was chosen for ease of computation. A suitable strategy was selected depending upon the contribution of each deletion towards an increase in lactate flux compared to the wild-type lactate flux. On the other hand, if the reaction list had greater than 30 reactions, only single reaction deletions were performed to identify potential knock-outs that improved lactate flux. For this *in silico* strain optimisation task, the COBRA Toolbox v3.0 functions ‘*removeRxns*’ and ‘*optimizeCbModel*’ were used for reaction deletions and FBA with optimisation of community biomass, respectively.

## 5 Data availability

All models used in this work and the codes used for our analysis are available at https://github.com/RamanLab/CAMP

### CRediT authorship contribution statement

**Maziya Ibrahim:** Conceptualization, Writing – original draft, Writing – review & editing. **Karthik Raman:** Conceptualization, Writing – review & editing.

## Declaration of Competing Interest

The authors declare that they have no known competing financial interests or personal relationships that could have appeared to influence the work reported in this paper.

## References

[1] Ye C., Zou W., Xu N., Liu L. (2014). Metabolic model reconstruction and analysis of an artificial microbial ecosystem for vitamin C production. J Biotechnol.

[2] Thuan N.H., Chaudhary A.K., Van Cuong D., Cuong N.X. (2018). Engineering co-culture system for production of apigetrin in *Escherichia coli*. J Ind Microbiol Biotechnol.

[3] Saini M., Lin L.-J., Chiang C.-J., Chao Y.-P. (2017). Synthetic Consortium of *Escherichia coli* for n-Butanol Production by Fermentation of the Glucose-Xylose Mixture. J Agric Food Chem.

[4] Jawed K., Yazdani S.S., Koffas M.AG. (2019). Advances in the development and application of microbial consortia for metabolic engineering. Metab Eng Commun.

[5] Ryu J.Y., Kim H.U., Lee S.Y. (2015). Reconstruction of genome-scale human metabolic models using omics data. Integr Biol (United Kingdom).

[6] Gu C., Kim G.B., Kim W.J., Kim H.U., Lee S.Y. (2019). Current status and applications of genome-scale metabolic models. Genome Biol.

[7] Kim M., Park B.G., Kim E.-J., Kim J., Kim B.-G. (2019). In silico identification of metabolic engineering strategies for improved lipid production in *Yarrowia lipolytica* by genome-scale metabolic modeling. Biotechnol Biofuels.

[8] Ravikrishnan A., Raman K. (2018).

[9] Stolyar S., Van Dien S., Hillesland K.L., Pinel N., Lie T.J., Leigh J.A. (2007). Metabolic modeling of a mutualistic microbial community. Mol Syst Biol.

[10] Chan S.H.J., Simons M.N., Maranas C.D., Price N.D. (2017). SteadyCom: Predicting microbial abundances while ensuring community stability. PLoS Comput Biol.

[11] Thommes M., Wang T., Zhao Q.i., Paschalidis I.C., Segrè D., Dutton R.J. (2019). Designing Metabolic Division of Labor in Microbial Communities. MSystems.

[12] Dusselier M., Van Wouwe P., Dewaele A., Makshina E., Sels B.F. (2013). Lactic acid as a platform chemical in the biobased economy: The role of chemocatalysis. Energy Environ Sci.

[13] Farah S., Anderson D.G., Langer R. (2016). Physical and mechanical properties of PLA, and their functions in widespread applications — A comprehensive review. Adv Drug Deliv Rev.

[14] Alves de Oliveira R., Komesu A., Vaz Rossell C.E., Maciel F.R. (2018). Challenges and opportunities in lactic acid bioprocess design—From economic to production aspects. Biochem Eng J.

[15] Juturu V., Wu J.C. (2016). Microbial production of lactic acid: the latest development. Crit Rev Biotechnol.

[16] Zhang Y., Vadlani P.V. (2015). Lactic acid production from biomass-derived sugars via co-fermentation of *Lactobacillus brevis* and *Lactobacillus plantarum*. J Biosci Bioeng.

[17] Cui F., Li Y., Wan C. (2011). Lactic acid production from corn stover using mixed cultures of *Lactobacillus rhamnosus* and *Lactobacillus brevis*. Bioresour Technol.

[18] Eş I., Mousavi Khaneghah A., Barba F.J., Saraiva J.A., Sant'Ana A.S., Hashemi S.M.B. (2018). Recent advancements in lactic acid production - a review. Food Res Int.

[19] Tarraran L., Mazzoli R. (2018). Alternative strategies for lignocellulose fermentation through lactic acid bacteria: The state of the art and perspectives. FEMS Microbiol Lett.

[20] Kristjansdottir T., Bosma E.F., Branco dos Santos F., Özdemir E., Herrgård M.J., França L. (2019). A metabolic reconstruction of *Lactobacillus reuteri* JCM 1112 and analysis of its potential as a cell factory. Microb Cell Fact.

[21] Choi H.S., Lee S.Y., Kim T.Y., Woo H.M. (2010). In silico identification of gene amplification targets for improvement of lycopene production. Appl Environ Microbiol.

[22] Spector M.P. (2009). Encyclopedia of Microbiology. Encycl Microbiol.

[23] Heinken A., Thiele I., Drake H.L. (2015). Anoxic conditions promote species-specific mutualism between gut microbes In Silico. Appl Environ Microbiol.

[24] Klitgord N., Segrè D., Papin J.A. (2010). Environments that Induce Synthetic Microbial Ecosystems. PLoS Comput Biol.

[25] Goldford J.E., Lu N., Bajić D., Estrela S., Tikhonov M., Sanchez-Gorostiaga A. (2018). Emergent simplicity in microbial community assembly. Science (80-).

[26] Zelezniak A., Andrejev S., Ponomarova O., Mende D.R., Bork P., Patil K.R. (2015). Metabolic dependencies drive species co-occurrence in diverse microbial communities. Proc Natl Acad Sci U S A.

[27] Machado D., Maistrenko O.M., Andrejev S., Kim Y., Bork P., Patil K.R. (2021). Polarization of microbial communities between competitive and cooperative metabolism. Nat Ecol Evol.

[28] Pacheco A.R., Moel M., Segrè D. (2019). Costless metabolic secretions as drivers of interspecies interactions in microbial ecosystems. Nat Commun.

[29] Thapa L.P., Lee S.J., Park C., Kim S.W. (2017). Production of L-lactic acid from metabolically engineered strain of *Enterobacter aerogenes* ATCC 29007. Enzyme Microb Technol.

[30] Mazumdar S., Clomburg J.M., Gonzalez R. (2010). *Escherichia coli* strains engineered for homofermentative production of D-lactic acid from glycerol. Appl Environ Microbiol.

[31] Zhu J., Shimizu K. (2005). Effect of a single-gene knockout on the metabolic regulation in *Escherichia coli* for D-lactate production under microaerobic condition. Metab Eng.

[32] Shen M.-H., Song H., Li B.-Z., Yuan Y.-J. (2015). Deletion of d-ribulose-5-phosphate 3-epimerase (RPE1) induces simultaneous utilization of xylose and glucose in xylose-utilizing *Saccharomyces cerevisiae*. Biotechnol Lett.

[33] Wu Y., Shen X., Yuan Q., Yan Y. (2016). Metabolic engineering strategies for co-utilization of carbon sources in microbes. Bioengineering.

[34] Chiang C, Lee HM, Guo HJ, Wang ZW, Lin L (2013). Systematic Approach To Engineer *Escherichia coli* Pathways for Co-utilization of a Glucose–Xylose Mixture. J. Agric. Food Chem.

[35] Khandelwal R.A., Olivier B.G., Röling W.F.M., Teusink B., Bruggeman F.J., Vera J. (2013). Community Flux Balance Analysis for Microbial Consortia at Balanced Growth. PLoS ONE.

[36] Zomorrodi A.R., Maranas C.D., Rao C.V. (2012). A multi-level optimization framework for the metabolic modeling and analysis of microbial communities. PLoS Comput Biol.

[37] John R.P., Madhavan Nampoothiri K. (2011). Co-culturing of *Lactobacillus paracasei subsp. paracasei* with a L*actobacillus delbrueckii subsp. delbrueckii* mutant to make high cell density for increased lactate productivity from cassava bagasse hydrolysate. Curr Microbiol.

[38] Garde A., Jonsson G., Schmidt A.S., Ahring B.K. (2002). Lactic acid production from wheat straw hemicellulose hydrolysate by *Lactobacillus pentosus* and *Lactobacillus brevis*. Bioresour Technol.

[39] Siezen R.J., van Hylckama Vlieg J.E.T. (2011). Genomic diversity and versatility of *Lactobacillus plantarum*, a natural metabolic engineer. Microb Cell Fact.

[40] Teusink B., Wiersma A., Molenaar D., Francke C., de Vos W.M., Siezen R.J. (2006). Analysis of growth of *Lactobacillus plantarum* WCFS1 on a complex medium using a genome-scale metabolic model. J Biol Chem.

[41] Xiong T., Peng F., Liu Y., Deng Y., Wang X., Xie M. (2014). Fermentation of Chinese sauerkraut in pure culture and binary co-culture with *Leuconostoc mesenteroides* and *Lactobacillus plantarum*. LWT - Food Sci Technol.

[42] Bertsch A., Roy D., LaPointe G. (2019). Enhanced exopolysaccharide production by *Lactobacillus rhamnosus* in co-culture with *Saccharomyces cerevisiae*. Appl Sci.

[43] Somkuti G.A., Steinberg D.H. (2010). Pediocin production in milk by *Pediococcus acidilactici* in co-culture with *Streptococcus thermophilus* and *Lactobacillus delbrueckii subsp. bulgaricus*. J Ind Microbiol Biotechnol.

[44] Cai J., Tan T., Chan S.H.J. (2020). Predicting Nash equilibria for microbial metabolic interactions. Bioinformatics.

[45] Noronha A., Modamio J., Jarosz Y., Guerard E., Sompairac N., Preciat G. (2019). The Virtual Metabolic Human database: Integrating human and gut microbiome metabolism with nutrition and disease. Nucleic Acids Res.

[46] Carr F.J., Chill D., Maida N. (2002). The lactic acid bacteria: A literature survey. Crit Rev Microbiol.

[47] Henry C.S., DeJongh M., Best A.A., Frybarger P.M., Linsay B., Stevens R.L. (2010). High-throughput generation, optimization and analysis of genome-scale metabolic models. Nat Biotechnol.

[48] Bauer E., Thiele I. (2018). From metagenomic data to personalized in silico microbiotas: predicting dietary supplements for Crohn’s disease. Npj Syst Biol Appl.

[49] Heirendt L., Arreckx S., Pfau T., Mendoza S.N., Richelle A., Heinken A. (2019). Creation and analysis of biochemical constraint-based models using the COBRA Toolbox vol 3.0. Nat Protoc.

[50] Mahadevan R., Schilling C.H. (2003). The effects of alternate optimal solutions in constraint-based genome-scale metabolic models. Metab Eng.

[51] Devika N.T., Raman K. (2019). Deciphering the metabolic capabilities of *Bifidobacteria* using genome-scale metabolic models. Sci Rep.

[52] Medlock G.L., Carey M.A., McDuffie D.G., Mundy M.B., Giallourou N., Swann J.R. (2018). Inferring Metabolic Mechanisms of Interaction within a Defined Gut Microbiota. Cell Syst.

[53] Magnúsdóttir S., Heinken A., Kutt L., Ravcheev D.A., Bauer E., Noronha A. (2017). Generation of genome-scale metabolic reconstructions for 773 members of the human gut microbiota. Nat Biotechnol.

[54] Ibrahim M., Raajaraam L., Raman K. (2021). Modelling microbial communities: Harnessing consortia for biotechnological applications. Computational and Structural Biotechnology Journal.

