# Supplementary material for: Two-species community design of lactic acid bacteria for optimal production of lactate
Source: Comput Struct Biotechnol J. 2021 Nov 9;19:6039–49. doi: 10.1016/j.csbj.2021.11.009 (PMC8605394; doi:10.1016/j.csbj.2021.11.009)
Supplement: Supplementary data 2 [file mmc2.docx]

**Supporting Information**

**
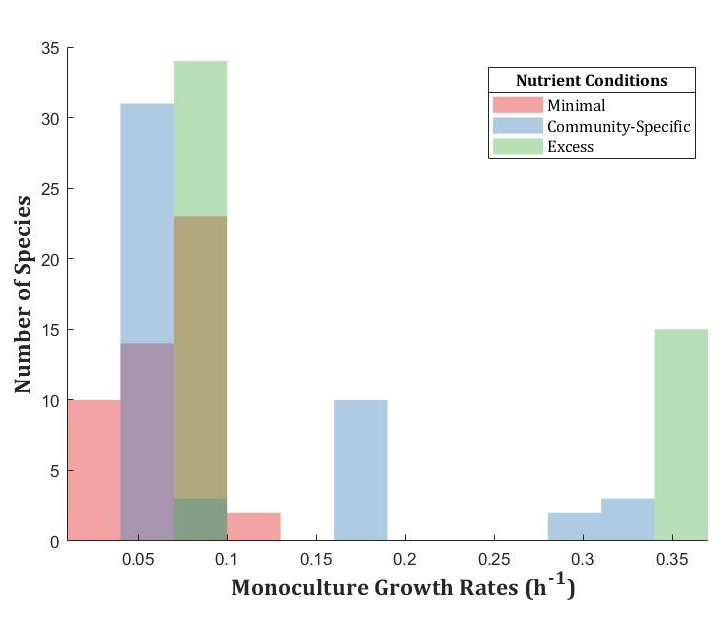
**

**S1 Fig.** **Histogram distribution of monoculture growth rates of all 49 species under three different nutrient conditions.** The highest growth rates (> 0.3 h^-1^) were observed in the community-specific and excess nutrient conditions.





**S2 Fig. Monoculture vs. Co-culture growth rates with excess nutrient uptake.** The heatmap depicts the change in the growth rate of an organism’s monoculture growth compared to when it is co-cultured with another species under excess nutrient uptake condition. A difference lesser than 10% of monoculture growth is regarded as a decrease. 838 non-viable pairs and the diagonal, which represents 49 monocultures, are depicted as white squares.





**S3 Fig. Monoculture vs. Co-culture growth rates with minimal nutrient uptake.** The heatmap depicts the change in the growth rate of an organism’s monoculture growth compared to when it is co-cultured with another species under minimal nutrient uptake condition. A difference greater than 10% of monoculture growth is considered an increase, lesser than 10% of monoculture growth is regarded as a decrease. 684 non-viable pairs and the diagonal, which represents 49 monocultures, are depicted as white squares.





**S4 Fig.** **Monoculture and Co-culture growth rates with minimal nutrient uptake.** The heatmap depicts the absolute values of the predicted growth rates of each organism in the community. Diagonal elements represent the monoculture growth rates of all 49 species. Non-viable communities are denoted in white squares.





**S5 Fig.** **Monoculture and Co-culture growth rates with community-specific nutrient uptake fluxes.** The heatmap depicts the absolute values of the predicted growth rates of each organism in the community. Diagonal elements represent the monoculture growth rates of all 49 species. Non-viable communities are denoted in white squares.





**S6 Fig.** **Monoculture and Co-culture growth rates in excess-nutrient condition.** The heatmap depicts the absolute values of the predicted growth rates of each organism in the community. Diagonal elements represent the monoculture growth rates of all 49 species. Non-viable communities are denoted in white squares.

**S1 File. The file consists of S1-S6 Tables.**

**S1 Table** lists the 49 Lactic Acid Bacteria GEMs used in this study. Growth rates and Lactate yields under three nutrient conditions with glucose and xylose as carbon sources.

**S2 Table.** Cross-fed metabolites found across all interaction types.

**S3 Table.** Community models whose observed lactate yield is 10-fold higher than the expected lactate yield in each nutrient state.

**S4 Table.** Reactions that have a 5-fold increase in flux among High-Lactate producer LAB communities.

**S5 Table.** Community models where a higher lactate flux than wild-type was observed upon deletion of one or two reactions shortlisted from FSEOF.

**S6 Table**. Metabolic Distances of all LAB communities.
